# *Ganoderma tsugae* Inhibits the SREBP-1/AR Axis Leading to Suppression of Cell Growth and Activation of Apoptosis in Prostate Cancer Cells

**DOI:** 10.3390/molecules23102539

**Published:** 2018-10-05

**Authors:** Shih-Yin Huang, Guan-Jhong Huang, Hsi-Chin Wu, Ming-Ching Kao, Wen-Chin Huang

**Affiliations:** 1 Graduate Institute of Biomedical Sciences, School of Medicine, China Medical University, Taichung 40402, Taiwan; nerissana777@gmail.com; 2 School of Chinese Pharmaceutical Sciences and Chinese Medicine Resources, College of Chinese Medicine, China Medical University, Taichung 40402, Taiwan; gjhuang@mail.cmu.edu.tw; 3 Department of Urology, China Medical University Hospital, Taichung 40402, Taiwan; wuhc@mail.cmuh.org.tw; 4 Department of Biological Science and Technology, College of Biopharmaceutical and Food Sciences, China Medical University, Taichung 40402, Taiwan; mckao@mail.cmu.edu.tw; 5 Research Center for Chinese Herbal Medicine, China Medical University, Taichung 40402, Taiwan; 6 Chinese Medicine Research Center, China Medical University, Taichung 40402, Taiwan

**Keywords:** AR, *Ganoderma tsugae*, lipogenesis, prostate cancer, SREBP-1

## Abstract

Recent research suggests that the activation of lipid biosynthesis (lipogenesis) is linked with prostate cancer (PCa) malignancy. Sterol regulatory element-binding protein-1 (SREBP-1) is a key transcriptional regulator controlling lipogenesis. Moreover, androgen receptor (AR) has been well defined to play an important role in lethal PCa aggressiveness from androgen-responsive to castration-resistant status. In this study, we showed that the quality-assured *Ganoderma tsugae* ethanol extract (GTEE), a Chinese natural and herbal product, significantly inhibited expression of SREBP-1 and its downstream genes associated with lipogenesis in PCa cells. Through inhibiting SREBP-1, GTEE reduced the levels of intracellular fatty acids and lipids in PCa cells. Importantly, GTEE also downregulated the expression of AR and prostate-specific antigen (PSA) in both androgen-responsive and castration-resistant PCa cells. By blocking the SREBP-1/AR axis, GTEE suppressed cell growth and progressive behaviors, as well as activating the caspase-dependent apoptotic pathway in PCa cells. These data provide a new molecular basis of GTEE for the development of a potential therapeutic approach to treat PCa malignancy.

## 1. Introduction

Cancer progression is the underlying cause of death in prostate cancer (PCa) patients. One of the main clinical challenges is that PCa progresses from androgen-responsive to androgen-refractory/castration-resistant prostate cancer (CRPC), which is incurable. Androgen receptor (AR), a master androgenic hormone-activated transcription factor, plays a key role in the regulation of PCa growth, survival, and deadly CRPC progression [1,2]. Androgen deprivation therapy (ADT), blocking the androgen/AR signaling pathway, is a first-line therapy in clinical intervention in PCa patients. However, most PCa patients who receive ADT suffer severe adverse effects and eventually relapse with lethal CRPC progression. Therefore, there is an urgent need to develop novel and effective treatments to prevent and cure this fatal aggressiveness.

Sterol regulatory element-binding proteins (SREBPs; SREBP-1 and SREBP-2) mainly control the expression of genes associated with lipogenesis, cholesterogenesis, and lipid/cholesterol homeostasis [3,4]. SREBPs would be able to interact with sterol regulatory element (SRE) *cis*-acting elements that are found in the promoter regions of genes encoding enzymes for the de novo biosynthesis of fatty acids, lipids, and cholesterol [3,5,6]. Specifically, SREBP-1 transcriptionally activates genes that are involved in lipogenesis, such as fatty acid synthase (FASN; a rate-limiting enzyme and a check point for lipogenesis), whereas SREBP-2 mediates cholesterol biosynthesis through the regulation of HMG CoA reductase (HMGCR; a rate-limiting enzyme and a check point for cholesterogenesis) [3,5,6]. We previously reported that SREBP-1 induced PCa cell growth and progression through the concerted activation of the metabolic and signaling networks involving lipogenesis, AR, and oxidative stress [7]. In addition, SREBP-1 regulated AR expression through transcriptionally activation in PCa cells [7,8]. Importantly, overexpression of SREBP-1 protein was associated with aggressive pathological features and CRPC progression in PCa patients [7,9]. These findings provide a rationale for targeting SREBP-1 and its regulated downstream axis as an attractive therapeutic approach to eliminating PCa aggressiveness.

Traditional Chinese medicine is a healing and herbal system, which has been established a medical foundation for a long history. *Ganoderma tsugae* (GT), a Chinese herbal product, is a restricted species of *Lingzhi* that is cultivated in Taiwan, and it has been shown to exhibit antioxidant activity, and it is applied to treat cardiovascular and allergic diseases [10,11]. Our laboratory previously demonstrated that an ethanol extract of GT (GTEE) displayed anti-proliferative effects on human cancer cells [12,13,14,15]. However, the clinical benefits and the molecular basis of GTEE in PCa malignancy remain unknown.

The aim of this study is to reveal and evaluate the molecular mechanisms and the therapeutic efficacy of a Chinese herbal medicine, GTEE, in PCa cells, including LNCaP (androgen-responsive) and C4-2 (castration-resistant) cells. GTEE inhibited the expression of SREBP-1 and FASN in LNCaP and C4-2 cells. By inhibiting genes associated with lipogenesis, GTEE reduced the amounts of intracellular fatty acid and lipid accumulation in PCa cells. Furthermore, GTEE decreased the expression of AR and prostate-specific antigen (PSA), an AR downstream target gene, in both LNCaP and C4-2 cells. GTEE also suppressed cell growth and aggressive behaviors, as well as inducing the caspase-dependent apoptotic pathway in PCa cells. Taken together, these results provide an innovative molecular basis of GTEE in PCa cells, and targeting the SREBP-1/AR axis by GTEE could be a promising approach for the treatment of malignant PCa. 

## 2. Results

### 2.1. GTEE Inhibits the Expression of SREBP-1 and Its Downstream Associated Genes in PCa Cells

**Figure 1:**
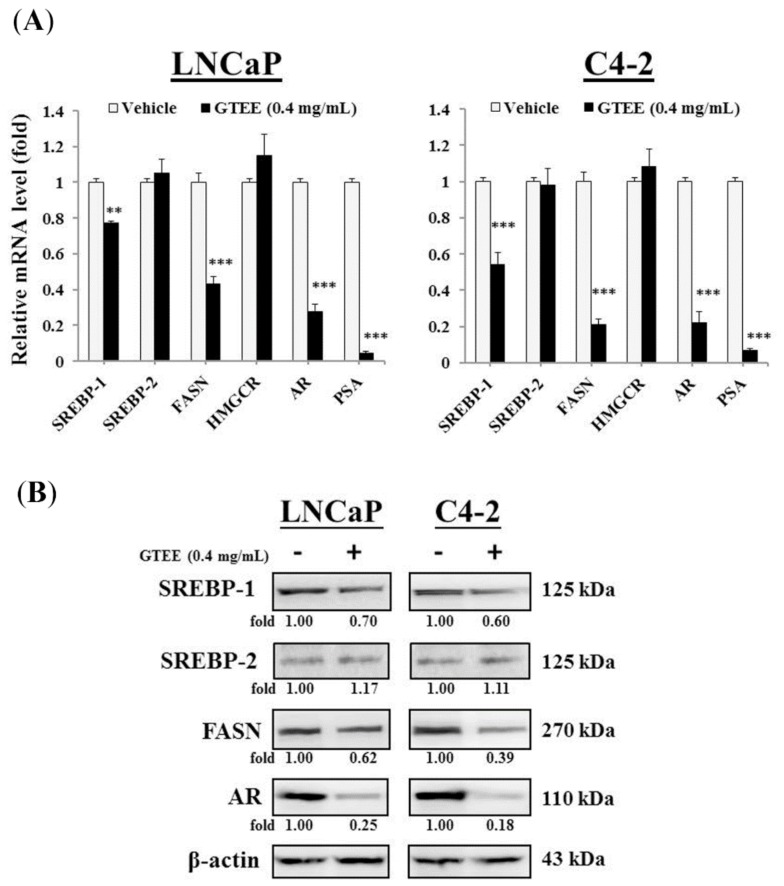
*Ganoderma tsugae* ethanol extract (GTEE) inhibits the expression of SREBP-1 and its downstream related genes in prostate cancer (PCa) cells. (**A**) GTEE significantly inhibited the mRNA expression of SREBP-1, FASN, AR, and PSA but not SREBP-2 and HMGCR in both LNCaP and C4-2 PCa cells determined by quantitative Reverse Transcription-Polymerase Chain Reaction (qRT-PCR) analysis. The relative mRNA level (fold) was assigned as 1.0 in vehicle-treated cells. Data were normalized to β-actin and represented as the mean ± SD of three independent duplicate experiments. ** *p* < 0.01, *** *p* < 0.001. (**B**) GTEE suppressed the protein levels of SREBP-1, FASN, and AR, but not SREBP-2 in LNCaP, and C4-2 cells assayed by Western blot analysis. β-actin was used as a loading control. The protein bands were scanned and quantified using ImageJ software. The relative level (fold) of protein expression with the vehicle treatment and normalized to β-actin was assigned as 1.00.

To investigate whether GTEE inhibits SREBP-1/lipogenesis and the AR axis in PCa cells, which play important roles in PCa development, survival, and progression [7,8,16,17], we performed quantitative Reverse Transcription-Polymerase Chain Reaction (qRT-PCR) and Western blot analyses to determine the expression of genes that are associated with SREBPs and AR. As shown in Figure 1A, GTEE decreased the mRNA expression of SREBP-1 and FASN in both LNCaP and C4-2 cells. However, GTEE did not significantly change the expression of SREBP-2 and HMGCR in PCa cells, which mainly controlled cholesterogenesis. We also examined whether GTEE affected AR and PSA expression in these AR-positive PCa cells, because we previously reported that SREBP-1 transcriptionally regulated AR expression [7,8]. By inhibiting SREBP-1 expression, GTEE decreased the mRNA expression of AR and its downstream target genes, PSA, in LNCaP and C4-2 cells (Figure 1A). Fitting with the effects of GTEE on mRNA expression, the protein levels of SREBP-1, FASN, and AR, but not SREBP-2 were also decreased by GTEE in LNCaP and C4-2 cells (Figure 1B). Collectively, the data of qRT-PCR and Western blot analyses suggest that GTEE inhibited the expression of SREBP-1 and its downstream associated genes, including FASN and AR, in PCa cells.

### 2.2. GTEE Reduces the Levels of Intracellular Fatty Acid and Lipid Accumulation in PCa Cells

**Figure 2:**
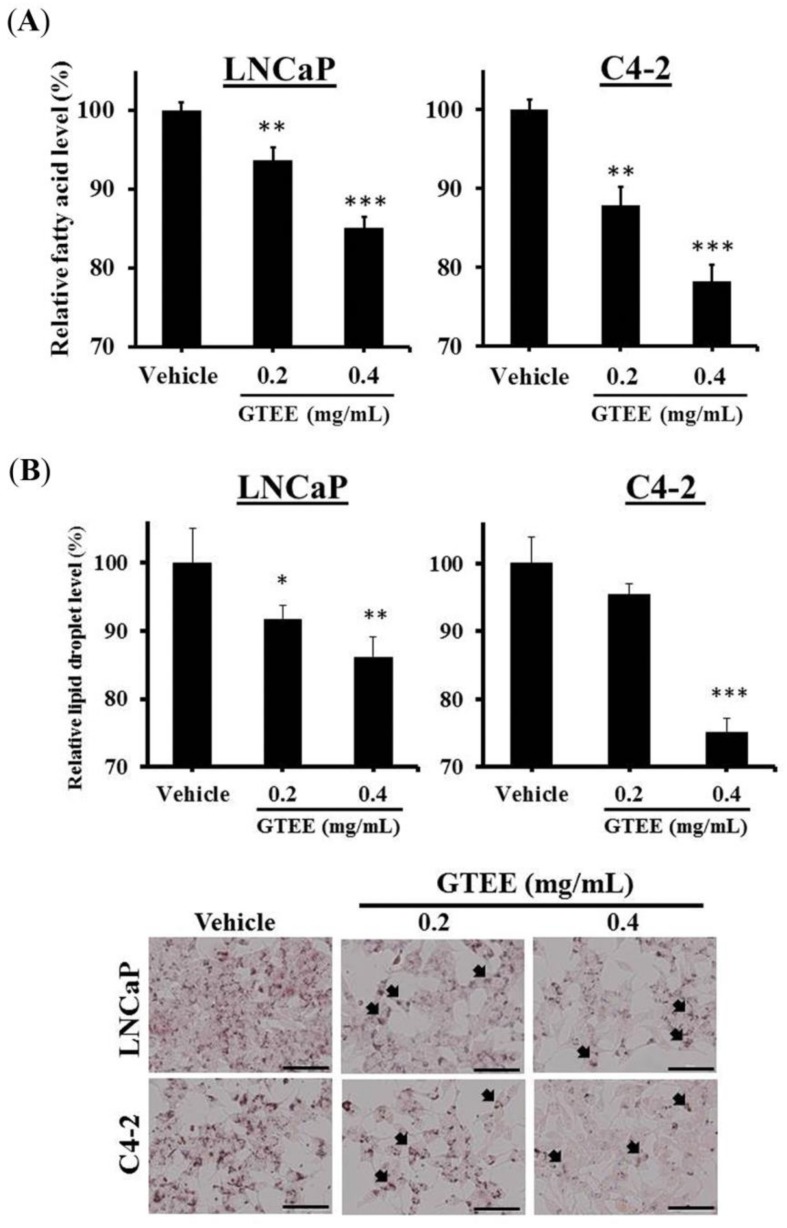
GTEE reduces the amounts of intracellular fatty acid and lipid accumulation in PCa cells. (**A**) LNCaP and C4-2 cells were treated with vehicle (0.3% ethanol) or GTEE (0.2 or 0.4 mg/mL) for 24 h. The levels of intracellular fatty acid were determined by a fatty acid quantification kit. The relative fatty acid level (%) was assigned as 100% in vehicle-treated PCa cells. Data were shown as the mean ± SD of three independent experiments. ** *p* < 0.01, *** *p* < 0.001. (**B**) GTEE decreased the amounts of lipid droplet accumulation assayed by the Oil Red O staining in LNCaP and C4-2 cells at 24 h. The relative lipid droplet level (%) was assigned as 100% in vehicle-treated cells. Data were shown as the mean ± SD of three independent experiments. * *p* < 0.05, ** *p* < 0.01, *** *p* < 0.001. The arrowheads indicate lipid droplets in PCa cells (bottom panel). Scale bars = 100 µm.

Because GTEE inhibited the expression of key genes (SREBP-1 and FASN) linked with lipogenesis, we subsequently performed quantification and staining assays to determine the changes of the intracellular fatty acid and lipid levels in PCa cells caused by GTEE. As shown in Figure 2A, the amounts of intracellular fatty acids were significantly decreased in GTEE-treated LNCaP and C4-2 cells in a dose-dependent pattern compared to a vehicle group. Furthermore, the lipid droplet accumulation was determined by the Oil Red O staining and quantification. The lipid droplet levels of GTEE-treated LNCaP and C4-2 cells were also decreased in comparison with vehicle-treated cells (Figure 2B). The results of lipogenesis assays indicate that GTEE led to a decrease in the levels of intracellular fatty acid and lipid accumulation in LNCaP and C4-2 cells through the inhibition of SREBP-1 and FASN (Figure 1).

### 2.3. GTEE Suppresses the Cell Growth and In Vitro Progression of PCa Cells

**Figure 3:**
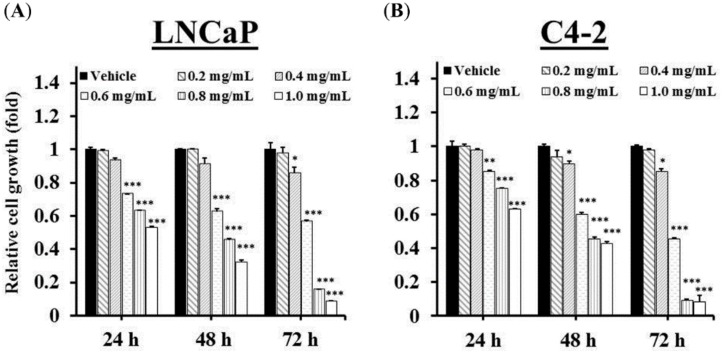
GTEE suppresses the cell growth of PCa cells. (**A**) LNCaP and (**B**) C4-2 cells were treated with vehicle (0.3% ethanol) or GTEE (0.2, 0.4, 0.6, 0.8 or 1.0 mg/mL) for 24, 48 and 72 h. Cell growth was determined by MTS assay. The relative cell growth (fold) was assigned as 1.0 in vehicle-treated cells at each time point. Data were shown as the mean ± SD of three independent experiments. * *p* < 0.05, ** *p* < 0.01, *** *p* < 0.001.

To assess the potential for biological consequences elicited by GTEE (Figure 1 and Figure 2), LNCaP and C4-2 cells were treated with various concentrations of GTEE followed by functional analyses, including cell growth and in vitro migration and invasion assays. We first determined the growth of PCa cells using a MTS assay. As shown in Figure 3A,B, GTEE suppressed the growth of LNCaP and C4-2 cells at varying concentrations (0.2, 0.4, 0.6, 0.8 and 1.0 mg/mL) and at varying times (24, 48 and 72 h). The half maximal inhibitory concentrations (IC_50_) of GTEE for LNCaP and C4-2 cells were 0.746 and 0.735 mg/mL (48 h), respectively.

**Figure 4:**
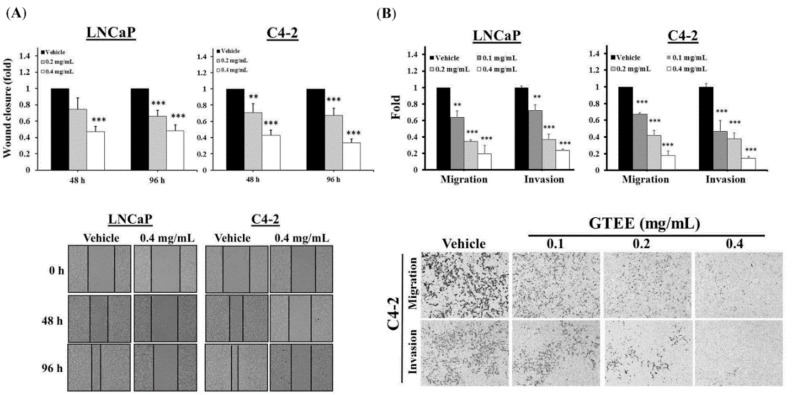
GTEE inhibits the in vitro progression of PCa cells. (**A**) LNCaP and C4-2 cells were treated with vehicle (0.3% ethanol) or GTEE (0.2 or 0.4 mg/mL). Wound closure was determined by migratory distance at 48 and 96 h. Data were shown as the mean ± SD of three independent experiments. ** *p* < 0.01, *** *p* < 0.001 (top panel). Representative images of wound closure in LNCaP and C4-2 cells treated with vehicle or GTEE (0.4 mg/mL) were shown (bottom panel). (**B**) The migration and invasion of LNCaP and C4-2 cells treated with vehicle (0.3% ethanol) or GTEE (0.1, 0.2 or 0.4 mg/mL) were performed by the Boyden chamber method. Data represented the mean ± SD of three separate experiments. ** *p* < 0.01, *** *p* < 0.001 (top panel). Representative images of the migration and invasion of C4-2 cells treated with vehicle or GTEE were shown (bottom panel).

Next, the effects of GTEE on the migratory and invasive potentials of PCa cells were determined. A wound healing method was used to evaluate the migratory ability. As shown in Figure 4A, GTEE treatment led to a significant inhibition of wound closure in both LNCaP and C4-2 cells. Besides, the Boyden chamber method was also used to determine migration and invasion in PCa cells that were affected by GTEE. GTEE (0.1, 0.2 and 0.4 mg/mL) significantly decreased the capabilities of migration and invasion of LNCaP and C4-2 cells in a concentration-dependent pattern (Figure 4B and Figure S1). These biofunctional results suggest that GTEE suppressed cell growth and in vitro progression in LNCaP and C4-2 cells.

### 2.4. GTEE Induces Apoptosis through the Caspase-Dependent Pathway in PCa Cells

**Figure 5:**
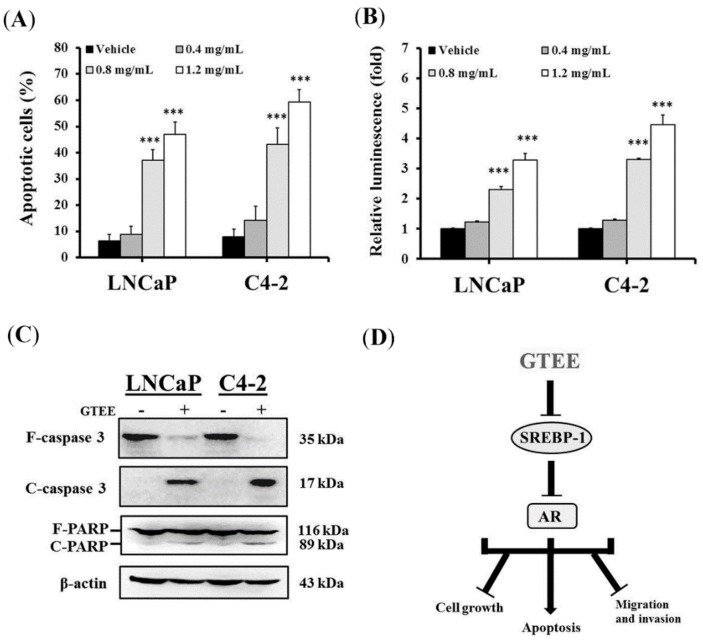
GTEE induces apoptosis through the caspase-dependent pathway in PCa cells. (**A**) After 24 h treatment with the vehicle (0.3% ethanol) or GTEE (0.4, 0.8 or 1.2 mg/mL), apoptotic cells (%) of LNCaP and C4-2 were determined by Annexin V-FITC staining analysis using flow cytometry. Data represented the mean ± SD of triplicate experiments. *** *p* < 0.001. (**B**) GTEE increased the caspase-3/7 activity in LNCaP and C4-2 cells with a dose-dependent manner. Luminescence of the caspase-3/7 activity was measured by an enzymatic activity assay. The relative luminescence (fold) was assigned as 1 in vehicle-treated cells. Results were shown as the mean ± SD of three independent experiments. *** *p* < 0.001. (**C**) Western blot analysis of caspase-3 and PARP proteins in PCa cells treated with vehicle (0.3% ethanol) or GTEE (1.2 mg/mL) for 20 h. GTEE greatly decreased full length (F)-caspase-3 as well as increased cleaved (C)-caspase-3 and C-PARP in LNCaP and C4-2 cells. β-actin was used as a loading control. (**D**) A schematic model of GTEE blocked the SREBP-1/AR axis to further suppress cell growth, migration, and invasion, and induce apoptosis in PCa cells was proposed.

We observed that many attached LNCaP and C4-2 cells on cultured dishes were floating/dying during treatment with high concentrations of GTEE, such as 0.8 or 1.2 mg/mL. Therefore, we next determined apoptotic death in PCa cells caused by GTEE. Flow cytometry-based annexin V-FITC staining analysis, caspase enzymatic activity assay, and Western blot analysis of caspase and PARP proteins were conducted to evaluate whether GTEE induced the caspase-dependent apoptotic pathway in PCa cells. The results of Annexin V-FITC staining analysis showed that GTEE increased the percentages of apoptotic cells in LNCaP and C4-2 cells with a concentration-dependent manner after 24 h treatment (Figure 5A). Subsequently, the caspase-3/7 enzymatic activity and the expression of caspase-3 and PARP proteins were examined. As shown in Figure 5B, GTEE significantly increased the caspase-3/7 activity in LNCaP and C4-2 cells in a dose-dependent pattern (0.8 and 1.2 mg/mL). Moreover, the results of Western blot analysis showed that GTEE greatly decreased the expression of full length (F)-caspase-3 (35 kDa) as well as increased the expression of cleaved (C)-caspase 3 (17 kDa) and C-PARP (89 kDa) in both LNCaP and C4-2 cells (Figure 5C). Collectively, these data suggest that GTEE induces the caspase-dependent apoptotic death in PCa cells.

## 3. Discussion

Activation of lipogenesis in cancer cells has been shown to be triggered by increased needs for the components of cell lipid bilayer membranes, and promotion of the signaling transduction mediated by cell membranes during uncontrolled cell division, growth, and tumor aggressiveness [18,19,20]. Overexpression of a master transcription regulator for lipogenesis, SREBP-1, was associated with aggressive pathologic features, including CRPC progression, and poor clinical outcomes in human PCa [7]. Therefore, targeting aberrant de novo fatty acid/lipid biosynthesis linked to SREBP-1 represents a new and attractive therapeutic strategy to treat PCa malignancy. In this study, we first discovered that GTEE would be able to suppress the genotypes (Figure 1) and phenotypes (Figure 2) of lipogenesis in both LNCaP (androgen-responsive) and C4-2 (castration-resistant) cells. Through the inhibition of SREBP-1 and FASN genes (Figure 1), determined by qPCR and Western blot analyses, GTEE decreased the levels of intracellular fatty acid and lipid accumulation (Figure 2) in PCa cells. Intriguingly, FASN has been reported to be a metabolic oncogene [21] and it is involved in PCa progression [9,22]. Furthermore, GTEE significantly suppressed cell growth, migration, and invasion in PCa cells. Indeed, it has been demonstrated that silencing of SREBP-1 caused a decrease in SREBP-1 downstream lipogenic genes, including FASN, inhibition of cell growth and aggressive behaviors, and induction of programmed death in PCa [23], endometrial [24], and ovarian [25] cancer cells. Moreover, the ablation of SREBP-1 expression led to induced endoplasmic reticulum stress, reactive oxygen species (ROS) accumulation, and apoptosis in glioblastoma and breast cancer cells [26]. Consistent with our previous findings [7], SREBP-1 was associated with oxidative stress and ROS production in PCa cells. Although we demonstrated that GTEE interrupted the SREBP-1-regulated metabolic pathway and suppressed PCa cell growth and progression in the present study, the molecular mechanism by which GTEE inhibits SREBP-1 expression in PCa cells remains unclear. In order to determine the molecular basis, an additional experiment was performed using a promoter-luciferase reporter vector of SREBP-1. As shown in Figure S2, GTEE significantly decreased the promoter-luciferase activity of SREBP-1 in LNCaP and C4-2 cells. Based on the results of qPCR, Western blotting (Figure 1) and the promoter reporter assays, GTEE blocking SREBP-1 in PCa cells is mediated through transcriptional regulation.

AR is referred to as one of the most significant protein factors for PCa, even in lethal CRPC aggressiveness, and it is still the primary focus of new drug design. Up-regulation of AR expression and activity has been well defined to promote PCa development, survival, and CRPC progression [1,2,27]. The therapeutic strategies of PCa treatment have been developed to target AR via a blockade of AR nuclear translocation [27,28], silencing of AR gene expression [8,29,30], including the full length or splice variants of AR, or interruption of the interaction between AR and its co-factors, as well as their downstream functions [27,28,31]. In this study, we revealed that GTEE significantly inhibited the expression of AR mRNA and protein in LNCaP and C4-2 cells. The molecular basis of AR expression inhibited by GTEE could be due to down-regulation of SREBP-1, because we previously demonstrated that SREBP-1 transcriptionally activated AR expression through binding of a SREBP-1 *cis*-acting element located in the 5′ flanking AR promoter region [8]. Besides, GTEE also reduced PSA expression in LNCaP and C4-2 cells. PSA is one of the downstream target genes of AR and it has been well used to diagnose PCa disease [32,33] as a clinically important serum biomarker for PCa. Taken together, through the inhibition of SREBP-1, GTEE impaired AR and PSA expression in PCa cells. This could be exploited for a better therapeutic application by co-targeting aberrant lipogenesis and the AR/PSA signaling pathway using GTEE in PCa.

GTEE also displayed the induction of apoptosis in PCa cells. Apoptosis is a biological process of programmed cell death that occurs in embryonic development, normal cell turnover, and cancer cell treatment. Many anti-cancer drugs have been reported to show efficacy on the induction of the apoptotic pathway in tumor cells [13,23,34]. Caspase-3 is a major factor that converges the mitochondrial-regulated and the death receptor-regulated apoptotic pathways in cells [35]. Upon the activation of caspase-3 enzymatic activity, the downstream substrates are cleaved, including PARP, and this leads to apoptotic cell death. Our data showed that GTEE-induced apoptosis through activation of caspase-3/7 enzymatic activity in PCa cells in a dose-dependent pattern (Figure 5B). Besides, GTEE increased protein expression of cleaved-caspase 3 and cleaved-PARP in LNCaP and C4-2 cells (Figure 5C). The activation of apoptosis-associated proteins by GTEE is responsible for the concomitant execution phase of programmed cell death in PCa cells. These findings suggest that GTEE would be able to kill PCa cells via the caspase-dependent pathway. Additionally, GTEE displayed no obvious cytotoxicity in a mouse model bearing human ovarian tumors [12]. This result implicates that GTEE may not affect normal cells in vivo, and it could be considered as a low toxic/safe therapeutic agent. Previous studies showed that several triterpenes isolated from *Ganoderma lucidum* inhibited cell proliferation and induced apoptosis in PCa cells [36,37]. We are currently attempting to purify and identify the ingredient(s) from GTEE, which exerts the apoptotic functions through blockade of the SREBP-1/AR axis in PCa cells. Potentially, the functional factors in GTEE or in the *Ganoderma* family will be applied as attractive new medicines for PCa treatment.

In conclusion, these results demonstrated for the first time that: (1) GTEE, a traditional Chinese medicine, inhibited the expression of SREBP-1, FASN, and AR in both androgen-responsive and castration-resistant PCa cells; (2) GTEE decreased the amounts of intracellular fatty acid and lipid in PCa cells; and 3) blockade of the SREBP-1/AR axis by GTEE resulted in significant inhibition of cell growth, migration and invasion, as well as induction of apoptosis via the caspase-associated pathway in PCa cells (Figure 5D). An additional study will be warranted to evaluate the anti-cancer efficacy of GTEE in animal models bearing PCa tumors in the near future. Taken together, the data support the possibility that GTEE, a natural and herbal product, can be developed as an effective pharmacologic strategy for the treatment of PCa malignancy.

## 4. Materials and Methods

### 4.1. PCa Cell Lines and Cell Culture

PCa cell lines, LNCaP (androgen-responsive) and C4-2 (androgen-independent /castration-resistant) cells, were kindly provided by Dr. Leland W.K. Chung (Cedars-Sinai Medical Center, Los Angeles, CA, USA) [38]. LNCaP and C4-2 cells were cultured in RPMI 1640 medium (Thermo Fisher Scientific/GIBCO, Waltham, MA, USA) supplemented with 10% fetal bovine serum (Thermo Fisher Scientific/HyClone), 100 U/mL penicillin, and 100 μg/mL streptomycin in a humidified 37 °C incubator with 5% CO_2_.

### 4.2. Preparation of GTEE

GT was provided by the Luo-Gui-Ying Fungi Agriculture Farm, Taoyuan, Taiwan. The powder of the GT fruiting body (30 g) was soaked in ethanol (3000 mL) and shaken for 24 h on a rotating shaker. After centrifugation, the supernatant was filtered through a filter paper (Whatman No.1, Cat. No. 1001-110) and the residues were extracted by ethanol twice, as mentioned above. The filtrates were harvested and subjected to concentration under reduced pressure to produce a brown gel-like GT extract (GTEE). Subsequently, GTEE was prepared as a stock solution with ethanol solvent (200 mg/mL) and stored at −80 °C until use. Furthermore, the quality control of GTEE was assessed and validated using both bioresponse fingerprint (PhytomicsQC platform) [39] as well as chemical fingerprint (HPLC and ESI-MS) [13] analyses, as described previously.

### 4.3. Quantitative Reverse Transcription-Polymerase Chain Reaction (qRT-PCR)

Total RNAs from PCa cells treated with GTEE or vehicle (0.3% ethanol) were isolated by RNeasy mini Kit (Qiagen, Valencia, CA, USA) and converted into cDNA using a SuperScript III First-Strand Synthesis Kit (Thermo Fisher Scientific/Life Technologies). Quantitative polymerase chain reaction was performed using the SYBR Green PCR Master mix and an ABI 7500 Fast Real-Time PCR System (Applied Biosystems, Grand Island, NY, USA). Data were normalized to β-actin as an internal reference and represented as the average ratio of triplicates. The oligonucleotide primer sets for qPCR, including SREBP-1, SREBP-2, FASN, HMGCR, AR, PSA, and β-actin were listed in Table S1.

### 4.4. Western Blot Analysis

The cell lysates were prepared from PCa cells treated with GTEE or vehicle (0.3% ethanol) using PRO-PREP Protein Extraction Solution (iNtRON technology, South Korea) with protease inhibitors added. The protein concentrations of the cell lysates were assayed using a Pierce™ BCA Protein Assay Kit (Thermo Fisher Scientific). For Western blotting, 50 µg of protein extract was loaded into the sodium dodecyl sulfate polyacrylamide gel electrophoresis (SDS-PAGE) gel and transferred onto polyvinylidene difluoride (PVDF) membranes. After blocking by 5% non-fat milk in PBST (PBS with Tween-20) buffer for 1 h at room temperature, PVDF membranes were incubated with primary antibodies for overnight at 4 °C, followed by incubation with a horseradish peroxidase (HRP)-conjugated secondary antibody. The reactive signals were visualized using an Enhanced Chemiluminescence Kit (Amersham Biosciences, Arlington Heights, IL, USA). Subsequently, the reactive protein bands were scanned and quantified using ImageJ software. Primary antibodies were used as follows: anti-SREBP-1, anti-FASN, anti-AR (Santa Cruz Biotechnology, Dallas, TX, USA), anti-SREBP-2 (Abcam, Cambridge, MA, USA), anti-caspase 3 (Novus Biologicals, Littleton, CO, USA), anti-PARP (GeneTex, Irvine, CA, USA), and anti-β-actin (Millipore, Burlington, MA, USA). 

### 4.5. The Fatty Acid Level and Lipid Droplet Accumulation Analyses

The levels of fatty acids in PCa cells were determined by a Fatty Acid Quantification Kit (MBL International Corporation, Woburn, MA, USA) according to the manufacturer’s instructions. Lipid droplet accumulation was assayed by an Oil Red O staining method as previously described [7,23]. Oil Red O staining images were examined and recorded by a phase contrast microscope. For quantification, Oil Red O retained in cells were extracted by 100% isopropanol and optical absorbance at 500 nm was measured, normalized by the total cell numbers.

### 4.6. Cell Growth and In Vitro Progression Assays

For cell growth assay [34], LNCaP and C4-2 cells were plated on 96-well plates (10,000 cells/well) and treated with various concentrations of GTEE or vehicle (0.3% ethanol). Cell growth was assayed by MTS assay (Promega Corp. Madison, WI, USA) according to the manufacturer’s instructions. In vitro cell migration or invasion [17,23] was determined by Boyden chambers pre-coated with nothing (for migration assay) or with growth factor-depleted Matrigel matrix (BD Bioscience, San Jose, CA, USA; for invasion assay). PCa cells (8 × 10^4^ cells/well) were seeded into the inside of the pre-coated upper chambers and treated with GTEE or vehicle (0.3% ethanol). After incubation for 48 h, a crystal violet staining method was used to measure the numbers of migrated or invading cells affected by GTEE. Additionally, a wound healing assay was performed as previously described [13]. 

### 4.7. Flow Cytometric Analysis

For analysis of apoptosis [34], PCa cells treated with GTEE or vehicle (0.3% ethanol) were stained using an Annexin V-FITC Apoptosis Detection Kit I (BD Biosciences) according to the manufacturer’s instructions. The amounts (%) of apoptotic cells were determined by flow cytometry and analyzed by the FACS Express v2.0 software.

### 4.8. Statistical Analysis

All data were analyzed at least three individual experiments by using a two-tailed unpaired Student’s *t* test for comparison of independent means. *P* values of less than 0.05 were considered to be statistically significant.

## Supplementary Materials

The following materials are available online. Figure S1: Representative images of the migration and invasion of LNCaP cells treated with vehicle or GTEE. Figure S2: The promoter-luciferase reporter activity of SREBP-1 was inhibited by GTEE in PCa cells. Table S1: The oligonucleotide primer sets for qPCR.
