# Supplementary material for: Ganoderma tsugae Inhibits the SREBP-1/AR Axis Leading to Suppression of Cell Growth and Activation of Apoptosis in Prostate Cancer Cells
Source: Molecules. 2018 Oct 5;23(10):2539. doi: 10.3390/molecules23102539 (PMC6222511; doi:10.3390/molecules23102539)
Supplement: Supplementary file 1 [file molecules-23-02539-s001.zip › Supplementary Files/Figure S1.pdf]

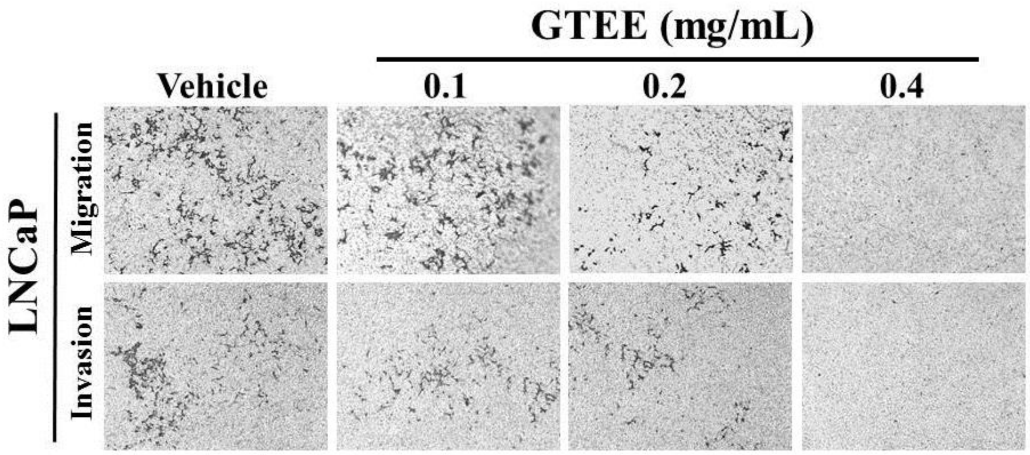

Figure S1. Representative images of the migration and invasion of LNCaP cells treated with vehicle or GTEE were shown.
