# Supplementary material for: Ganoderma tsugae Inhibits the SREBP-1/AR Axis Leading to Suppression of Cell Growth and Activation of Apoptosis in Prostate Cancer Cells
Source: Molecules. 2018 Oct 5;23(10):2539. doi: 10.3390/molecules23102539 (PMC6222511; doi:10.3390/molecules23102539)
Supplement: Supplementary file 1 [file molecules-23-02539-s001.zip › Supplementary Files/Figure S2.pdf]

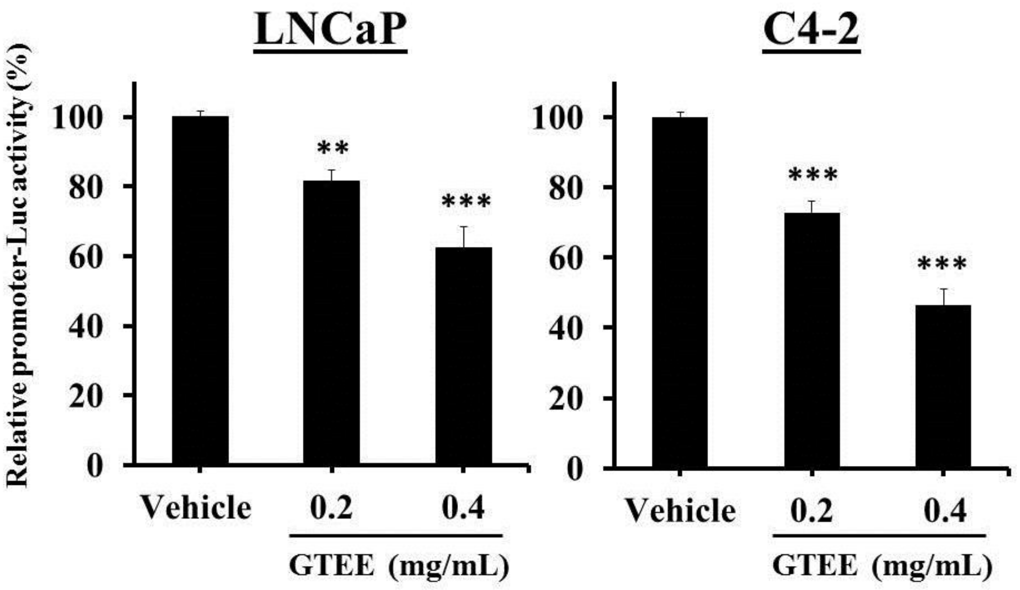

Figure S2. The promoter-luciferase (Luc) reporter activity of SREBP-1 (5′-flanking promoter region of SREBP-1: -1477/+158; obtained from GeneCopoeia) was inhibited by GTEE (0.2 and 0.4 mg/mL) in PCa cells. The relative promoter-Luc activities were shown as the mean ± SD of three independent experiments. $^{**}P < 0.01$, $^{***}P < 0.001$.
