# Supplementary material for: Ganoderma tsugae Inhibits the SREBP-1/AR Axis Leading to Suppression of Cell Growth and Activation of Apoptosis in Prostate Cancer Cells
Source: Molecules. 2018 Oct 5;23(10):2539. doi: 10.3390/molecules23102539 (PMC6222511; doi:10.3390/molecules23102539)
Supplement: Supplementary file 1 [file molecules-23-02539-s001.zip › Supplementary Files/Supplementary Table 1.pdf]

Table S1. The oligonucleotide primer sets for qPCR.

Gene	Forward (5'-3')	Reverse (5'-3')
SREBP-1	TCAGCGAGGCGGCTTTGGAGCAG	CATGTCTTCGATGTCGGTCAG
SREBP-2	CCCCTGACTTCCCTGCTGCA	GCGCGAGTGTGGCCGGATC
FASN	CGGTACGCGACGGCTGCCTG	GCTGCTCCACGAACTCAAACACCG
HMGCR	GTCATTCCAGCCAAGGTTGT	GGGACCACTTGCTTCCATTA
AR	ATGGCTGTCATTCAGTACTCCTGGA	AGATGGGCTTGACTTTCCCAGAAAG
PSA	ATGTGGGTCCCGGTTGTCTTCCTCACCCTGTC	ATGTGGGTCCCGGTTGTCTTCCTCACCCTGTC
β-actin	CAAGGCCAACCGCGAGAAGATGAC	GCCAGAGGCGTACAGGGATAGCACA
